# Fecal virome of paraguayan hairy dwarf porcupine (*Coendou spinosus*, Cuvier, 1823) in Rio de Janeiro, Brazil

**DOI:** 10.1007/s42770-026-01963-3

**Published:** 2026-05-20

**Authors:** Thamiris dos Santos Miranda, Matheus Augusto Calvano Cosentino, Filipe Romero Rebello Moreira, Francine Bittencourt Schiffler, Amanda Coimbra, Ricardo Mouta, Gabriel Medeiros, Déa Luiza Girardi, Victor Wanderkoke, Monique Lima, Thiago Henrique de Oliveira, Talitha Mayumi Francisco, Flávio Landim Soffiati, Suelen Sanches Ferreira, Carlos Ramon Ruiz-Miranda, Marcelo Alves Soares, Mirela D’arc, André Felipe Andrade dos Santos

**Affiliations:** 1 Laboratório de Diversidade e Doenças Virais, Universidade Federal do Rio de Janeiro (UFRJ), Rio de Janeiro, RJ Brazil; 2 Laboratório de Ciências Ambientais, Universidade Estadual do Norte Fluminense Darcy Ribeiro (UENF), Campos dos Goytacazes, Rio de Janeiro, RJ Brazil; 3 Associação Mico-Leão-Dourado, Silva Jardim, Rio de Janeiro, RJ Brazil; 4 Programa de Oncovirologia, Instituto Nacional de Câncer, Rio de Janeiro, RJ Brazil

**Keywords:** Free-living host, Feces, High-throughput sequencing, Brazilian wild rodent, Viral metagenomics

## Abstract

The Paraguayan hairy dwarf porcupine (*Coendou spinosus*, Cuvier, 1823) is a rodent species (Rodentia, Erethizontidae) widely distributed in the Brazilian Atlantic forest. However, little is known about their viral diversity. In this study, we aimed to evaluate, using high-throughput sequencing (HTS), the virome of the feces of seven healthy adult free-living porcupines from Silva Jardim, Rio de Janeiro, Brazil. Total viral nucleic acid was extracted and used for the library preparation for HTS using the Illumina MiSeq platform. The bioinformatics pipeline included quality control, with taxonomic assignments by Kraken2 and Diamond. Unclassified RNA viruses were investigated for viral genome characterization. A total of 41 viral families were classified, of which only seven were validated by both taxonomic analysis tools, including bacteriophages, vertebrate viruses, and unclassified RNA viruses. The most abundant bacterial reads identified belonged to the phylum Proteobacteria. In addition, in-depth analyses of RNA viruses revealed the presence of the *Tombusviridae* family, a group of plant-infecting viruses possibly associated with the host’s diet. This study provides new insights into the fecal virome of Paraguayan hairy dwarf porcupines, contributing to the knowledge of microbial diversity in Erethizontidae and supporting non-invasive virome studies in wildlife.

## Introduction

Paraguayan hairy dwarf porcupines (*Coendou spinosus*, Cuvier, 1823) are nocturnal, arboreal rodents of the family Erethizontidae, inhabiting tropical forests in Bolivia, Brazil, Venezuela, and the Guianas. They are herbivores with sharp spines (modified hairs) and prehensile tails [1]. Habitat loss, together with their ecological adaptability and frequent presence near human settlements, may facilitate the bidirectional transmission of zoonotic pathogens among wildlife, domestic animals, and humans [2]. Porcupines serve as reservoirs for several pathogenic agents, including helminths, bacteria, protozoa, and especially viruses, some of which are associated with emerging human infections [3–8]. Particular attention has been given to *Brazilian porcupine poxvirus* (BPoPV) (*Poxviridae*) [7], *Adenovírus 1* (*Adenoviridae*) [8], and *Erethizon dorsatum papillomavirus 1* (EdPV1) and *2* (EdPV2) (*Papillomaviridae*) [6]; all associated with disease in porcupines.

The porcupine’s role as a host for diverse viral agents, combined with its increasing proximity to urban areas, highlights the need to characterize its virome, which remains largely unexplored. The advent of high-throughput sequencing (HTS) technologies has markedly accelerated the discovery and identification of both known and novel viruses over the last decade. This expansion has improved our understanding of the virome in various vertebrate hosts, including rodents, which are among the main reservoirs of zoonotic viruses [9–11]. Given the rise in frequency of epidemics caused by zoonotic viruses, increasing research and surveillance efforts have been undertaken to characterize the viromes of wild fauna [11]. Therefore, this study aimed to characterize the fecal virome of free-living Paraguayan hairy dwarf porcupine (*Coendou spinosus*) individuals using samples from an area designated for the conservation of golden lion tamarins (*Leontopithecus rosalia*) in the State of Rio de Janeiro, Brazil.

## Materials and methods

###  Sample collection

**Fig. 1:**
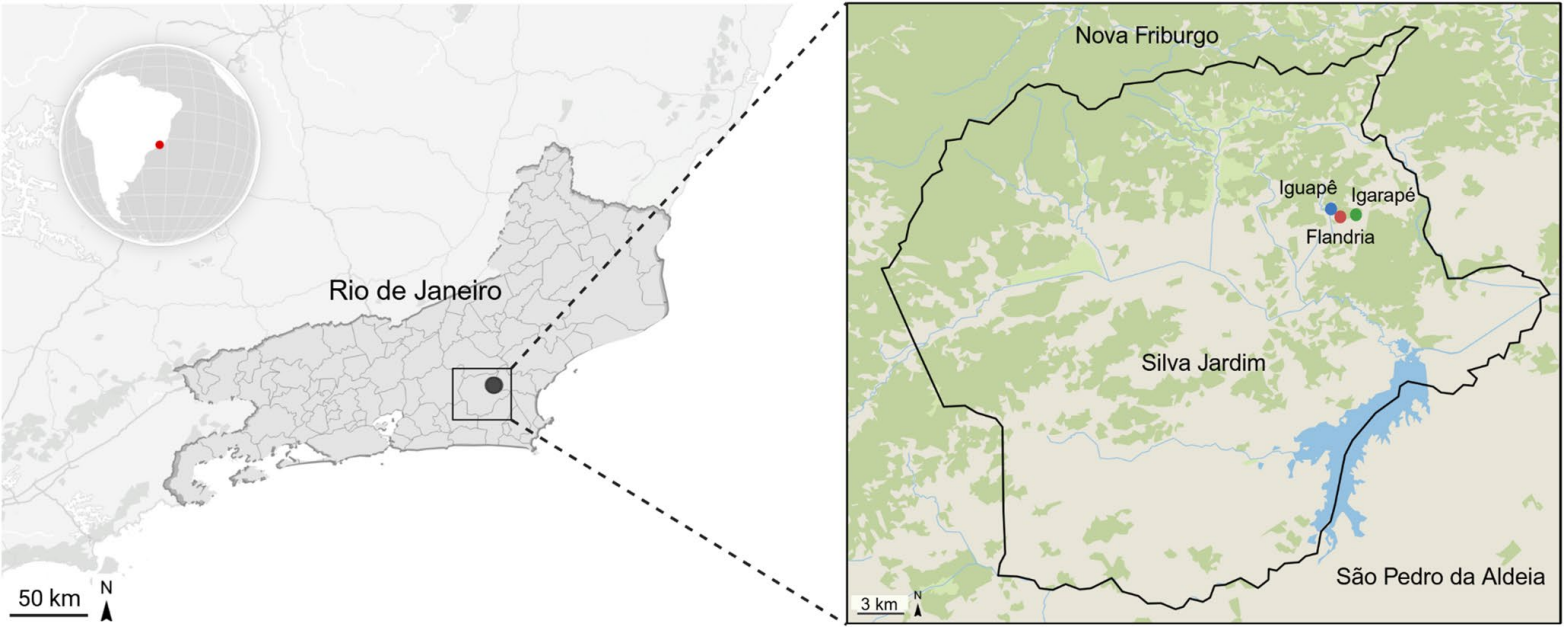
Sampling sites of *Coendou spinosus* in Atlantic Forest fragments, Silva Jardim, Rio de Janeiro, Brazil. Capture points: Iguapê Farm (blue; 22°30’20.7"S, 42°19’24.3"W), Igarapé farm (green; 22°30’25.7"S, 42°18’34.3"W), and flandria farm (red; 22°30’34.2"S, 42°19’07.2"W). Coordinates (WGS84). Map created with datawrapper (www.datawrapper.de)

Fecal samples were collected from seven healthy adult Paraguayan hairy dwarf porcupines (*Coendou spinosus*) on three farms: Iguapê (CSP01, CSP03 and CSP09), Igarapé (CSP10, CSP11 and CSP13), and Flandria (CSP12), between May and July 2019, in Silva Jardim, Rio de Janeiro, Brazil (Fig. 1). The Igarapé farm is managed as an ecological park by the *Associação Mico Leão Dourado* (AMLD), while Iguapê and Flandria are within the protected *Área de Proteção Ambiental da Bacia do Rio São João/Mico-Leão-Dourado* (APA). Animals were manually restrained, placed in breathable bags, weighed, and anesthetized with two protocols: Ketamine (10 mg/kg) + Xylazine (0.5 mg/kg) + Azaperone (2 mg/kg); or, Ketamine (10 mg/kg) + Dexmedetomidine (0.025 mg/kg). Physiological parameters were monitored throughout. Fecal samples were aseptically collected immediately after defecation, with the central portion of the fecal bolus collected to minimize external and soil contamination, and then transferred and homogenized into sterile 50 mL tubes with RNAlater™ (1:1 ratio; Thermo Fisher Scientific, USA) to preserve nucleic acid integrity for subsequent molecular analysis. Samples were transported and kept at room temperature for periods ranging from 1 to 7 days prior to stored at −80 °C in the *Laboratório de Diversidade e Doenças Virais* (LDDV) of the *Universidade Federal do Rio de Janeiro* (UFRJ). All procedures were approved by the Brazilian Federal Authority for scientific purposes (protocols 67274-8 and 64635-5).

### Viral enrichment and nucleic acid extraction

Samples were thawed and subsequently re-homogenized by vortexing for 1–3 min until to form a uniform fecal-RNAlater suspension. Subsequently, 1 mL of each was transferred to an extraction bead tube (MP Biomedicals, USA) and subjected to mechanical disruption. After disruption, samples were centrifuged and the supernatant was transferred to a new tube, and to optimize sequencing, fecal samples were grouped into a single pool with 200 µL of each sample before filtration with a Millex-HV 0.45 µm filter (Merck & Co., USA). After filtration, for additional viral particle enrichment, samples were ultracentrifuged at 35,000 x g for 90 min at 4 °C, followed by pellet resuspension and incubation with DNases (Promega and Epicentre, USA), RNase A (Ambion, USA), and Benzonase (Sigma, USA) at 37 °C for 90 min to degrade unprotected nucleic acids outside viral particles. Viral RNA and DNA were then extracted using the QIAamp MinElute Virus Spin Kit (QIAGEN, Germany), following the manufacturer’s instructions with previously described modifications [12]. Complementary DNA (cDNA) synthesis was performed using the Superscript III First Strand Synthesis Supermix Kit (Thermo Fisher Scientific, USA), followed by second-strand synthesis with the Klenow fragment 3’−5’ exo (New England Biolabs Inc., USA), according to the manufacturer’s instructions. During laboratory processing, all materials and work surfaces were treated with nucleic acid–degrading cleaning solutions to prevent carryover contamination.

### High-throughput sequencing and data analysis

Total nucleic acids (DNA and cDNA) were used for library preparation with the Nextera XT DNA Sample Preparation Kit (Illumina Inc., USA). Sequencing was performed on the Illumina MiSeq platform (MiSeq Reagent Kit v2, 2 × 151 bp; 300 cycles) at the Department of Genetics, UFRJ. A custom bioinformatics pipeline [12] included quality control with Fastp v.0.20.1 [13]; *de novo* assembly with Meta-spades v3.15.3 [14]; taxonomic classification with Kraken2 [15] and Diamond v.2.0.14 [16]; and visualization with Krona v.2.7.1 [17] and Pavian v.1.2.1 [18]. To minimize index-hopping artifacts, a control strategy was implemented by including just viral families representing > 1% of the highest read count per family across libraries in the same cartridge, minimizing the risk of cross-contamination [19].

Unclassified RNA viruses were reanalyzed by comparing filtered reads against a local database of non-redundant (nr) viral proteins from NCBI (as of November 16, 2024) using Diamond. Assignments were validated through reference mapping in Geneious Prime v2024.2.2 (high-sensitivity, minimum mapping quality Q30). Open reading frames (ORFs) were predicted with NCBI’s ORFfinder and annotated using BLASTp [20].

###  Phylogenetic analysis

To characterize the putative novel *Tombusviridae* detected by HTS, sequences were compared against a dataset of tombus-like viruses [21]. This dataset included 357 viral sequences (282 TC-Tombus-LVs and 75 H-Tombus-LVs), 62 reference sequences of classified viruses, and 99 unassigned tombus-like viruses retrieved from GenBank (https://www.ncbi.nlm.nih.gov/genbank/). Amino acid sequences were visualized with Aliview v1.27 [22] and aligned using MAFFTv.7.525 [23]. Alignment regions with ≥ 10% gaps were removed with trimAl v.1.4 [24], reducing non-informative sites. A Maximum Likelihood phylogeny was inferred with IQ-Tree v.2.1.4 [25] under the VT + F+R10 substitution model. Branch support was calculated with 10,000 ultrafast bootstrap (UF-Boot) replicates [26] and 10,000 Shimodaira-Hasegawa approximate likelihood-ratio test (SH-aLRT) replicates [27]. The final tree was visualized and annotated with Figtree v1.4.4 [28]. Classification within *Tombusviridae* followed the International Committee on Taxonomy of Viruses (ICTV) guidelines [29].

## Results

The porcupine fecal virome analyzed was derived from a composite sample of seven adult individuals (two males and five females) from three farms in Silva Jardim, Rio de Janeiro. The library generated 2,109,411 raw reads, of which 949,294 (45%) passed quality filtering. Kraken2 classified 350,852 reads (37%) mostly Bacteria (192,280, 54.8%), followed by Eukarya (156,712, 44.7%) and viruses (344, 0.1%) (Supplementary Table I). After index-hopping filtering, 23 viral families were identified (Supplementary Table II).

**Fig. 2:**
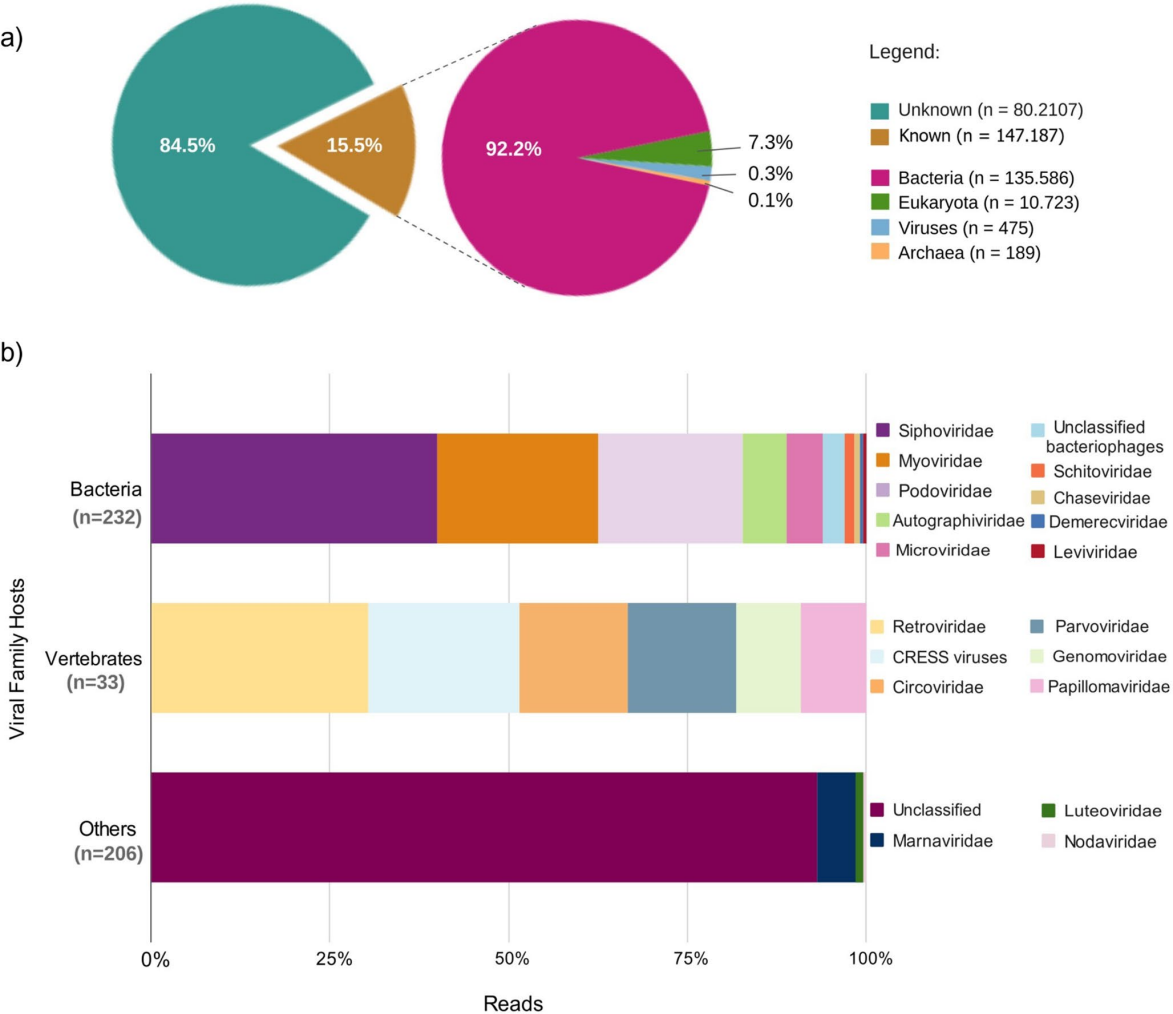
Taxonomic profile of the *Coendou spinosus* fecal virome (DIAMOND). (**a**) Classified and unclassified sequences by domain. (**b**) Viral family reads linked to vertebrate, bacterial, and other hosts

**Fig. 3:**
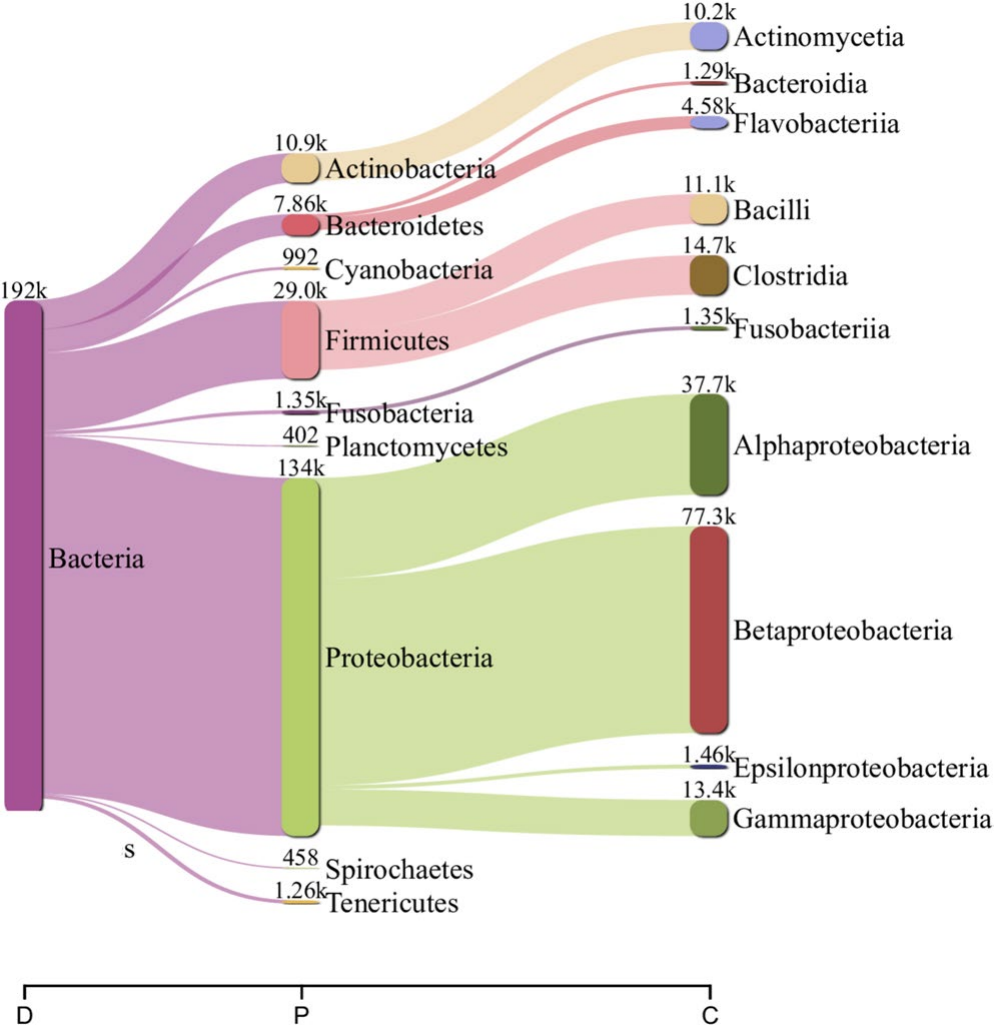
Sankey diagram of Kraken2 taxonomic classification of the *Coendou spinosus* fecal virome. Flow width reflects read abundance; labels indicate k-mer hits per node. Taxonomic ranks: D (domain), P (phylum), C (class)

Diamond assigned 147,187 reads (15.5%) from the NCBI nr database: Bacteria (135,586, 92.2%), Eukaria (10,723, 7.3%) and viruses (475, 0.3%) [Fig. 2a, Supplementary Table I]. Eighteen viral families were confirmed: mainly *Caudovirales* bacteriophages as *Siphoviridae* (93, 19.7%), *Myoviridae* (52, 11%), *Podoviridae* (47, 10%), and *Autographiviridae* (14, 3%) [Fig. 2b, Supplementary Table II]; vertebrate viruses included *Retroviridae* (10, 2.1%), CRESS virus (7, 1.5%), *Circoviridae* (5, 1.1%), *Parvoviridae* (5, 1.1%), *Genomoviridae* (3, 0.7%), and *Papillomaviridae* (3, 0.7%); and other families infected protozoa (*Marnaviridae* (11, 2.3%) and plants (*Luteoviridae* (2, 0.4%). *De novo* assembly yielded 4,107 contigs (125–2,067 nt). Only three were classified as *Siphoviridae*, *Podoviridae* and *Demerecviridae* by Kraken2, and two as *Retroviridae* and unclassified DNA virus by Diamond. Overall, 62.8% DNA viruses and 37.2% RNA viruses were sequenced. Only seven (38.8%) overlapped between Kraken2 and Diamond: *Siphoviridae*, *Myoviridae*, *Podoviridae*, *Autographiviridae*, *Schitoviridae*,* Demerecviridae* and *Papillomaviridae* (Supplementary Table II). Bacterial analysis showed Proteobacteria as most abundant (70% Kraken, 53% Diamond), followed by Firmicutes (15%, 36%), Actinobacteria (6%, 4%) and Bacteroidetes (4%, 4%) (Fig. 3).

**Fig. 4:**
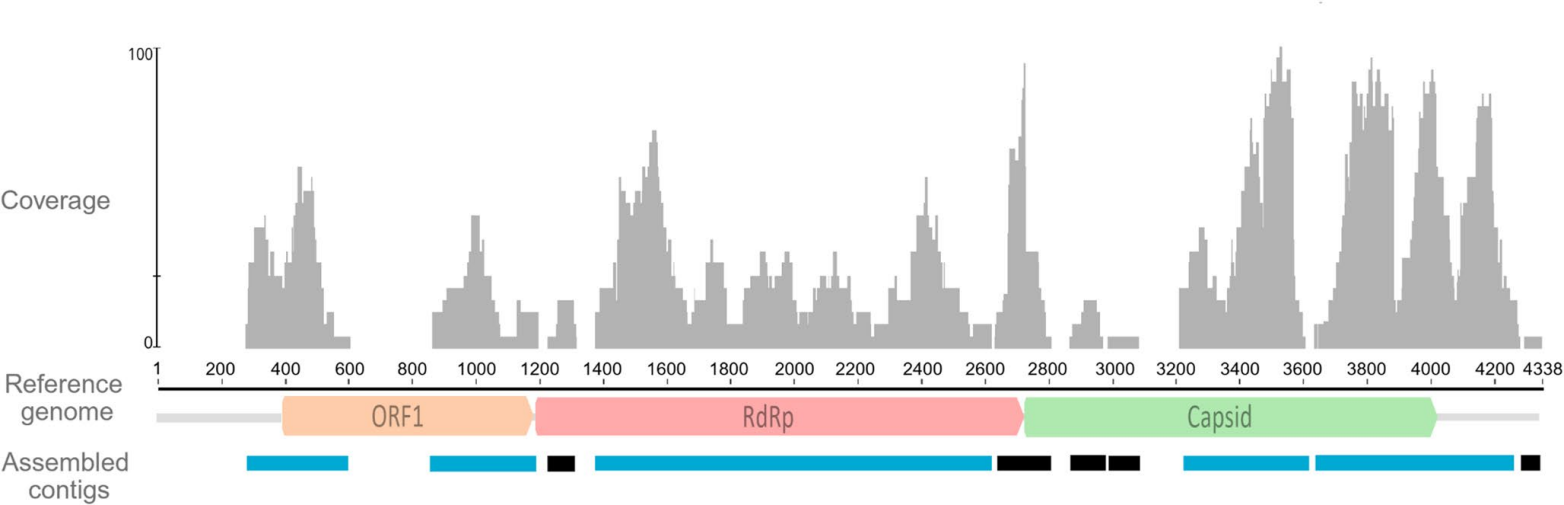
Reference mapping of filtered *Coendou spinosus* fecal virome reads in Geneious Prime (v2024.2). The 4,338 bp *Tolivirales sp.* genome (MR233-17E/2125; OR843652.1) was used as reference. Five of ten contigs (> 300 bp, blue) aligned with partial ORFs: ORF1 (P33-like), RdRp, and capsid. Fragments < 300 bp shown in black

Unclassified riboviruses were found by Kraken2 (22 reads, 6.4%) and Diamond (136, 28.9%) (Supplementary Table II). These were mapped to *Tolivirales sp. strain MR233-17E/2125* (TC tombus-like virus 32, 4,338 pb, OR843652.1), from the *Tombusviridae* family. A total of the 1,288 reads assembled into ten contigs, covering 87.8% of the reference genome (Fig. 4). BLASTn showed 97.4% identity with strain MR233-17E/2125.

**Fig. 5:**
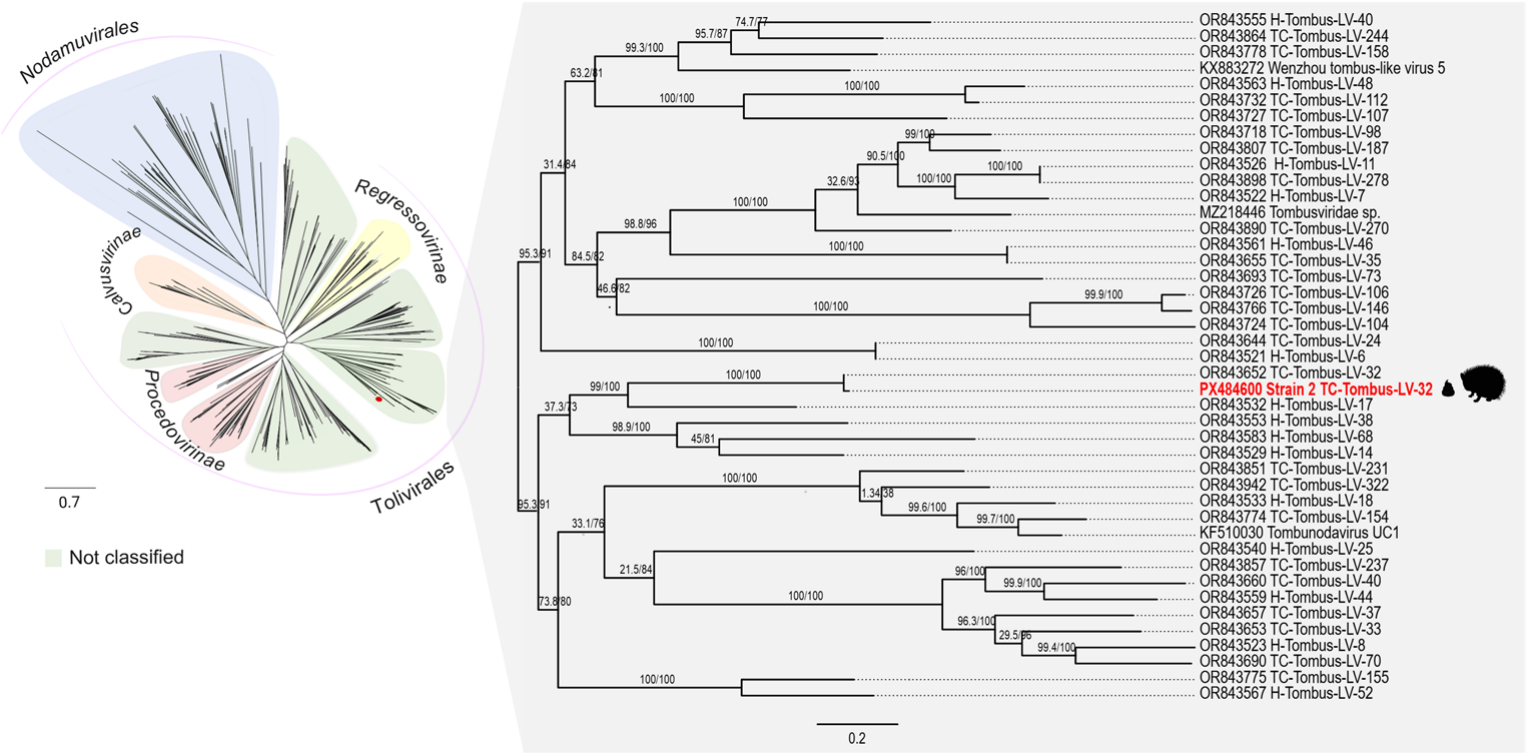
Maximum Likelihood phylogeny of tombus-like viruses based on 518 RdRp sequences (364 aa). Alignment by MAFFT v7.525; gaps ≥ 10% removed with trimAl v1.4. Tree inferred with IQ-TREE2 v2.1.4 (VT + F+R10; 10,000 ultrafast bootstrap and SH-aLRT). Visualized in FigTree v1.4.4. Scale bar: substitutions per site. Strain 2 TC-Tombus-LV-32 shown in red

Five contigs (> 300 bp, 329–1,245 bp) were analyzed with ORFfinder, representing three main ORFs: (a) ORF1, two fragments of tombusvirus P33-like protein (71–104 aa, 27%–40% coverage, 93–99% identity, 2e-50–5e-77 e-value, WRQ65314.1); (b) ORF2, a fragment of RNA-dependent RNA polymerase (RdRp) protein (413 aa, 53% coverage, 98.6% identity, 0.0 e-value, WRQ65315.1); (c) ORF3, two fragments of capsid structural protein (130–127 aa, 26–30% coverage, 83.9–97.7% identity, 5e-87–5e-56 e-value, WRQ65316.1). Phylogenetic analysis of the RdRp ORF confirmed clustering with TC-Tombus-LV-32 in the Tolivirales clade (Fig. 5). Following ICTV criteria (≥ 85% aa identity in RdRp/capsid), the virus was provisionally designated *Tolivirales sp. strain 2 MR233-17E/2125* (TC-Tombus-LV-32). As *Tombusviridae* infect plants, preliminary virome analysis also detected plant sequences (362 reads, 16 contigs) mainly from Fabales (family Fabaceae, legumes) (171 reads, 47.2%) (Supplementary Table III).

## Discussion

The wild porcupine may act as a reservoir for viral, bacterial, and fungal agents, including flaviviruses and poxviruses [3–8], as well as an uncharacterized microbiota, highlighting the need for its description and monitoring. Despite the low sequencing yield in the fecal virome (likely due to sample degradation, low viral load, or methodological limitations), several viral families were identified by metagenomic analysis. Overall, 41 viral families were detected using both taxonomic tools, with *Caudovirales* bacteriophages (*Siphoviridae*, *Myoviridae*, and *Podoviridae*) comprising 41%. As reported previously, *Caudovirales* were the predominant double-stranded DNA bacteriophages in mammalian fecal microbiomes [30, 31]. Variations in their abundance among mammals likely reflect differences in colonization patterns and gut bacterial dynamics, but may also be influenced by technical factors such as extraction efficiency, viral enrichment strategies, and sequencing biases [32]. Even with standardized protocols and strict procedures to minimize contamination, residual technical variability cannot be fully excluded and may contribute to the observed patterns.

Although viral metagenomics targets viral diversity, sequencing competition often yields more reads from other taxa [33]. In porcupine feces, bacterial reads accounted for 55%−92% of sequences. The microbiota was dominated by Proteobacteria (53–70%), followed by Firmicutes (15–36%), Actinobacteria (4–6%) and Bacteroidetes (4%). This differs from other rodents, such as Javan porcupines (*Hystrix javanica*) and capybaras (*Hydrochoerus hydrochaeris*), where Firmicutes predominate, followed by Bacteroidetes and a smaller proportion of Proteobacteria [5, 34]. However, these studies involved captive or anthropogenically influenced animals, which may not reflect wild microbiota. Still, it is important to emphasize that the predominance of Firmicutes, Bacteroidetes, and Proteobacteria in beavers, horses, rabbits, and koalas [35–38], suggests a common pattern among herbivores.

A high abundance (“bloom”) of Proteobacteria in the gut has been proposed as a marker of dysbiosis in humans and mice, however, this concept may not be directly applicable to wildlife species [39]. Studies in healthy wild rodents suggest that higher Proteobacteria levels may constitute a natural feature of the gut microbiota shaped by ecological or physiological adaptations, rather than a dysbiosis, particularly when compared to laboratory animals [40].

Proteobacterias include key gastrointestinal pathogens such as *Escherichia coli*, *Campylobacter jejuni*, *Klebsiella pneumoniae*,* Salmonella typhimurium*, and *Yersinia enterocolitica* [41]. Although Krona visualization allowed detection of sequences corresponding to *E. coli*, *C. jejuni*, and *K. pneumoniae*, all animals appeared clinically healthy, with no signs of gastrointestinal disease. However, as bacterial taxonomic classification was limited to the phylum level, it did not distinguish pathogenic from commensal bacteria. Targeted molecular approaches, such as species-specific or virulence gene–specific PCR, are needed to confirm their presence and pathogenic relevance. These analyses are also essential to clarify the potential role of porcupines as carriers of zoonotic bacteria at the wildlife–human interface, especially considering their proximity to human settlements.

Regarding viruses, only seven families were consistently identified by both taxonomic methods, including bacteriophages and one vertebrate family, *Papillomaviridae.* Members of this family, often causing benign or malignant epithelial lesions, including in porcupines [6, 42]. Notably, no epithelial lesions or other clinical abnormalities were observed at sampling, supporting the animals’ healthy status. As sequencing was performed on a single pooled sample, the prevalence, inter-individual variation and low-abundance or host-specific viruses could not be assessed; thus, future analyses of individual samples may enhance detection sensitivity.

A substantial proportion of unclassified riboviruses related to *Tombusviridae*, typically associated with plant hosts, may represent dietary-derived viruses, as reported in fecal viromes of various animals [31, 33, 43]. Similarly, metagenomic surveys of forest rodents in China identified novel plant viruses, with *Tombusviridae* among the most prevalent families [43]. Members of this family are single-stranded, positive-sense RNA viruses (~ 3.7–4.8 kb) [29], transmitted via soil or vectors such as arthropods or fungi [44, 45]. They include species infecting major crops, such sugarcane, with significant agricultural impact in regions such as Brazil [46–48]. Genomic analyses revealed *Tolivirales* sp. strain MR233-17E/2125 (TC-Tombus-LV-32), originally reported from the Teltow Canal, Berlin, Germany [21]. Its close phylogenetic relationship with the Brazilian strain suggests a common origin, possibly linked to global plant trade or environmental persistence. Porcupines may have acquired the virus by ingesting infected or contaminated vegetation, underscoring the role of anthropogenic activities in virus dissemination.

The likelihood of sequencing artifacts or environmental contamination is minimal, as multiple genomic regions of the *Tombusviridae* virus were sequenced and samples were directly collected from the animal using sterile material, without soil contact, supporting a dietary origin. Although the definitive host remains undetermined, metagenomic analysis detected plant signals predominantly from Fabaceae. This aligns with reports that *Coendou* porcupines in the Atlantic Forest have a frugivorous and folivorous diet, with preference for fruits and seeds of Fabaceae species [49]. Moreover, the known tropism of *Tombusviridae* for dicotyledonous plants, particularly Fabaceae [50], supports these plants as potential hosts.

In conclusion, this study provides the first assessment of the fecal virome of Paraguayan hairy dwarf porcupines (*Coendou spinosus*), revealing viral and bacterial diversity within Erethizontidae. Additionally, a diet-associated, unclassified group of RNA viruses was identified, highlighting the complexity of host–diet–virome interactions. These findings illustrate how metagenomic approaches expand knowledge of viral diversity in wildlife and support the use of fecal samples as non-invasive substrate for viral surveillance, monitoring, and detection of emerging or re-emerging viruses at the wildlife–environment interface without stressing the host. This approach is particularly relevant in conservation areas near human settlements, where early detection may aid both wildlife health management and public health preparedness.

## Data Availability

Raw sequencing reads are available under BioProject accession number PRJNA972708 and NCBI SRA accession number SRR24628734. Assembled contigs, taxonomic assignment files and Krona plots are available on the project GitHub page (link: https://github.com/lddv-ufrj/Porcupine_Virome). Tombusvirus sequences have been deposited in the NCBI database under the accession numbers PX484598–PX484602.
